# Detection Rate and Diagnostic Value of Optical Coherence Tomography Angiography in the Diagnosis of Polypoidal Choroidal Vasculopathy: A Systematic Review and Meta-Analysis

**DOI:** 10.1155/2019/6837601

**Published:** 2019-12-14

**Authors:** Yuelin Wang, Jingyuan Yang, Bing Li, Mingzhen Yuan, Youxin Chen

**Affiliations:** Department of Ophthalmology, Peking Union Medical College Hospital, Chinese Academy of Medical Sciences, Beijing 100730, China

## Abstract

**Purpose:**

This study aimed to evaluate the detection rate of polyps and branching vascular networks (BVNs) in polypoidal choroidal vasculopathy (PCV) by optical coherence tomography angiography (OCTA) and assess the sensitivity and specificity of OCTA in differentiating PCV from wet age-related macular degeneration (wAMD).

**Materials and Methods:**

We searched PubMed, EMBASE, Cochrane Library, and other sources. The detection rates of polyps and BVNs in observational studies and the sensitivity and specificity of PCV diagnosis from wAMD in diagnostic studies were extracted.

**Results:**

Twenty studies (573 eyes) were eligible. The combined detection rate of OCTA in PCV polyp lesion diagnosis was 0.67 (95% CI: 0.55–0.79), while that of BVNs was 0.86 (95% CI: 0.81–0.91). The detection rate of polyps was compared with that of BVNs in the same study, and the combined relative risk was 0.82 (95% CI: 0.72–0.92). The combined sensitivity of PCV diagnosis in wAMD patients using OCTA was 0.77 (95% CI: 0.55–0.90), combined specificity 0.84 (95% CI: 0.60–0.95), and area under the SROC curve 0.87 (95% CI: 0.84–0.90).

**Conclusion:**

OCTA has a high PCV polyp and BVN detection rate, and the detection rate of BVNs is higher than that of the polyp. OCTA has acceptable sensitivity and specificity for diagnosing PCV from wAMD. Thus, OCTA may be helpful for clinical diagnosis of PCV.

## 1. Introduction

Polypoidal choroidal vasculopathy (PCV) is a macular disease characterized by polypoidal-like dilated choroidal vessels surrounded by a branching vascular network (BVN) [1]. PCV was first proposed by Yannuzzi et al. in 1982 [2], and its pathogenesis has remained unclear thus far. PCV is often accompanied by pigment epithelium detachment, subretinal fluid, and other lesions; therefore, some scholars believe that PCV is a subtype of wet age-related macular degeneration (wAMD) [3]. However, due to the differences in clinical manifestations, pathogenesis, treatment, and prognosis between PCV and typical wAMD, distinguishing PCV from typical wAMD is necessary. Between these conditions, whether a polyp or BVN lesion is detected in the affected eye is an important identifier [4].

Currently, the commonly used PCV diagnostic method is indocyanine green angiography (ICGA). According to the EVEREST study [5], focal hyperfluorescent lesions appearing before 6 minutes on ICGA are necessary for the diagnosis of PCV. However, ICGA is an invasive operation; a contrast agent must be injected into the patient, and this agent may lead to an anaphylactic reaction. ICGA requires a professional operator, and it is not routinely performed in many parts of the world. Because of the limitations of ICGA, the use of other imaging examinations to diagnose PCV is currently a popular research area.

Optical coherence tomography angiography (OCTA) is a new imaging method to visualize the vasculature noninvasively [6]. The image-forming principle of OCTA is based on signals reflected from flowing blood cells in fundus vessels. At present, OCTA plays an important role in the field of fundus diseases, such as the diagnosis of wAMD, the discovery of choroidal neovascularization (CNV) in the fundus of high myopia, and the nonperfusion area of diabetic retinopathy.

In recent years, some studies have reported the detection rate of polyps and BVN lesions by OCTA and the sensitivity and specificity of the diagnosis of PCV from wAMD in patients. At present, the results among these studies are inconsistent, and no study has summarized and analyzed these data to draw a unified conclusion. Therefore, we performed a systematic review and meta-analysis to explore the detection rate of PCV lesions by OCTA and the diagnostic value of PCV from wAMD in patients.

## 2. Materials and Methods

This study applied the Cochrane Collaboration's Preferred Reporting Items for Systematic Reviews and Meta-Analyses (PRISMA) method for meta-analysis [7].

### 2.1. Study Selection

Observational studies and diagnostic tests related to the diagnosis of PCV by OCTA were included. Eligible observational studies met the following criteria: (1) ICGA-confirmed PCV cases (polypoidal hyperfluorescence on ICGA with or without abnormal BVN) and (2) OCTA was used for examination, and the detection rate of polyps or BVNs was reported. Diagnostic tests met the following requirements: (1) study subjects had wAMD eyes (OCT or any angiography suggesting drusen and choroidal neovascularization) and (2) OCTA and ICGA were separately used for detection, and diagnostic sensitivity and specificity were reported. Exclusion criteria were as follows: (1) eyes with other common eye diseases, such as pathological myopia, diabetic retinopathy, retinal artery, or venous occlusion; (2) low-vision eyes that cannot fix well to complete the OCTA examination; and (3) poor-quality OCTA image.

### 2.2. Search Strategy

We searched PubMed, Medline, EMBASE, and Cochrane Library from inception to March 1, 2019. We also searched other relevant resources for additional literature. No language restrictions were applied.

The search strategy was (OCTA OR optical coherence tomography angiography) AND (PCV OR polypoidal choroidal vasculopathy). One author (WYL) executed the search strategy, and another author (YJY) peer reviewed the strategy independently.

Two authors (WYL and YJY) independently reviewed titles and abstracts for inclusion, and full manuscripts were examined if necessary. A third researcher (CYX) participated in the discussion if the two authors disagreed.

### 2.3. Data Extraction

We extracted the name of the author, year of publication, type of study, type of OCTA, inclusion and exclusion criteria, proportion of treated PCV eyes, characteristics of participants, and outcomes from each study. The primary outcomes were (1) the detection rate of polyps or BVNs in PCV by OCTA examination and (2) the sensitivity and specificity of diagnosis of PCV by OCTA examination from wAMD eyes.

### 2.4. Risk of Bias

The quality of the included diagnostic studies was assessed by using the Quality Assessment of Diagnostic Accuracy Studies-2 (QUADAS-2) [8]. Each evaluator assessed the risk of bias, including patient selection, index test, reference standards flow, and timing, and other potential biases. Each criterion was assessed by scoring “low risk,” “high risk,” or “unclear risk.” Any disagreement was solved by discussion.

### 2.5. Statistical Analysis and Exploration of Heterogeneity

Statistical analysis was performed using Stata version 12 (Stata Corp, College Station, TX). The combined detection rate of polyps and BVNs was calculated from each article on a per lesion-based analysis using the following formula: detection rate = true positive/(true positive + false negative). The comparison between the detection rates of polyps and BVNs in the same study was reported with relative risk (RR). For diagnostic tests, we tabulated true positives, false negatives, false positives, and true negatives in PCV eyes diagnosed by OCTA and ICGA from wAMD eyes. We used an exact binomial rendition of the bivariate mixed-effects regression model for meta-analysis and modified one for synthesis of diagnostic test data [9].

We evaluated the heterogeneity among multiple studies by using the *I*^2^ method with the *χ*^2^ test to calculate the *P* value. If the homogeneity test showed *P* > 0.1 and *I*^2^ < 50%, which indicated a high homogeneity in designs between the included studies, we used a fixed-effect model (Mantel–Haenszel method) to combine the summary statistics. If a higher *I*^2^ value was shown, which indicated a high statistical heterogeneity between included studies, we used a random-effect model (DerSimonian–Laird method) to combine the summary statistics. All pooled data were presented with 95% confidence intervals (95% CIs). For publication bias evaluation, funnel plots and Egger's test were used.

## 3. Results and Discussion

### 3.1. Description of the Selected Studies

**Figure 1:**
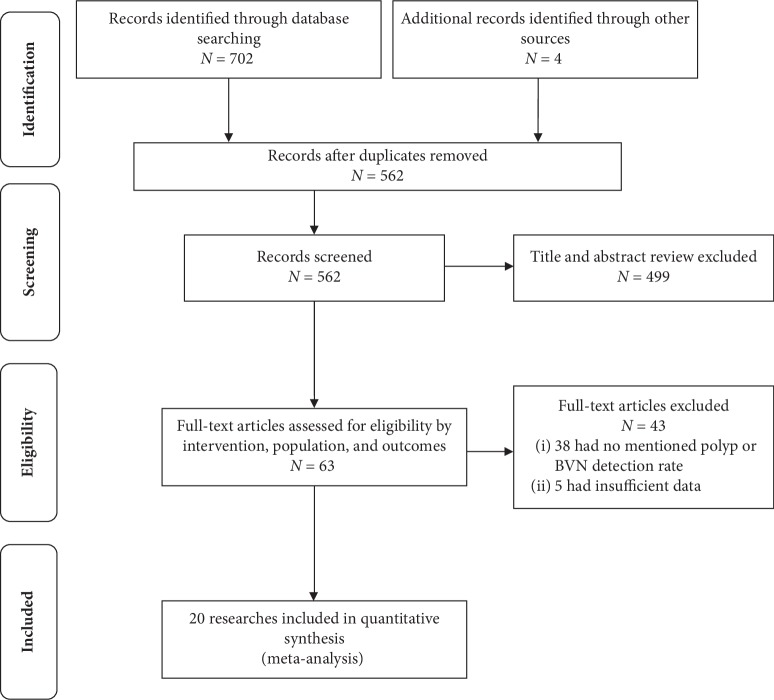
Flowchart of study selection. BVN indicates the branching vascular network.

We initially identified 706 articles. After excluding 643 records by screening the titles and abstracts, a total of 63 manuscripts were fully examined (see Figure 1). We ultimately included 20 studies [10–29] (640 eyes, 563 PCV eyes) for meta-analysis (see Table 1). We included 20 studies [10–29] reporting the polyp detection rate, 15 studies [10–12, 14, 15, 17–23, 25, 28, 29] reporting the BVN detection rate, and 4 articles [10, 11, 17, 21] (6 studies) reporting the sensitivity and specificity of OCTA and ICGA for diagnosing PCV from wAMD eyes. The literature quality evaluation is shown in [Supplementary-material supplementary-material-1] in Supplementary Materials.

### 3.2. Polyp and BVN Detection Rates

**Figure 2:**
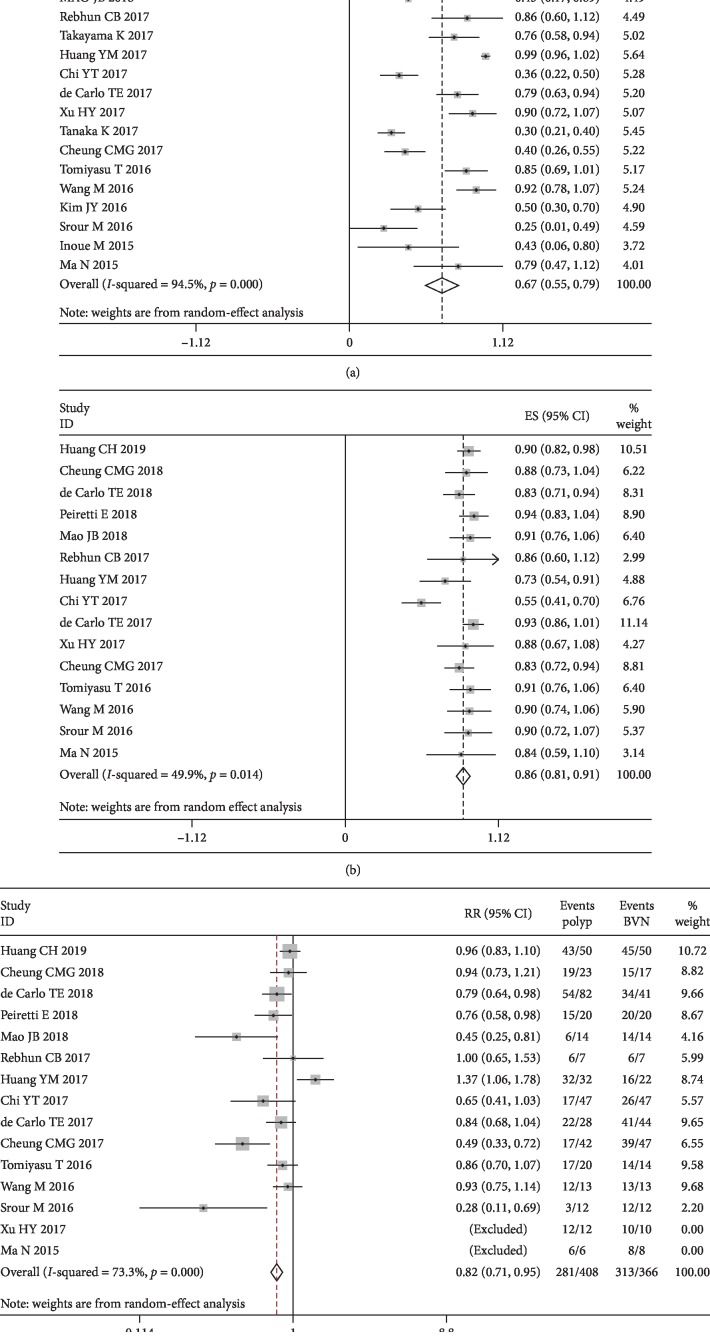
Forest plot showing the PCV lesion detection rate. The combined detection rate of polyps (a) and BVNs (b) and the relative risk (RR) of comparing the detection rates of polyps and BVNs (c). BVN: branching vascular network; CI: confidence interval; RR: relative risk. RR was calculated using a random-effect model (DerSimonian–Laird method).

The combined detection rate of polyps using OCTA was 0.67 (95% CI: 0.55–0.79; *I*^2^ = 94.5%) (see Figure 2(a)), and the detection rate of BVNs was 0.86 (95% CI: 0.81–0.91; *I*^2^ = 49.9%) (see Figure 2(b)). The funnel plots are shown in [Supplementary-material supplementary-material-1] in Supplementary Materials. Egger's regression intercepts for the detection rate of polyp and BVN pooling were −3.51 (95% CI: −6.84 to −1.71; *P*=0.003) and −1.23 (95% CI: −3.54 to 0.97; *P*=0.024), respectively. We also performed subgroup analysis. We analyzed the polyp and BVN detection rates by the subgroup using manual or automatic segmentation, SD or SS-OCT, and whether treated PCV was included. The results showed no significant difference between the subgroups (see Figures [Supplementary-material supplementary-material-1] and [Supplementary-material supplementary-material-1] in Supplementary Materials).

The combined polyp detection rate was compared with the BVN detection rate in the same study. The combined RR was 0.82 (D-L pooled RR; 95% CI: 0.71–0.95; heterogeneity: *P*=0.000; *I*^2^ = 73.3%) (see Figure 2(c)), suggesting that the detection rate of BVNs was higher than that of polyps when OCTA was used to diagnose PCV. The funnel plot is shown in [Supplementary-material supplementary-material-1] in Supplementary Materials. Egger's regression intercepts were −0.34 (95% CI: −2.09 to 1.53; *P*=0.741).

### 3.3. Sensitivity and Specificity of Diagnostic Tests

**Figure 3:**
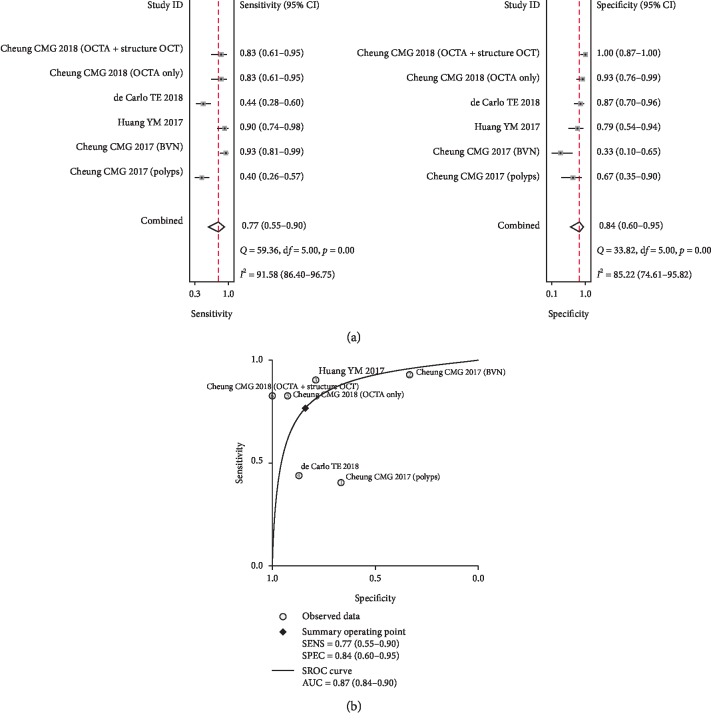
Forest plot and SROC curve showing the diagnostic value of OCTA in PCV diagnosis. Forest plot showing the combined sensitivity and specificity of OCTA in the diagnosis of PCV (a) and the SROC curve of OCTA in the diagnosis of PCV (b).

The combined sensitivity of OCTA for the diagnosis of PCV was 0.77 (95% CI: 0.55–0.90), the specificity was 0.84 (95% CI: 0.60–0.95), and the positive likelihood ratio was 4.8 (95% CI: 1.7–13.6). The ratio was 0.28 (95% CI: 0.13–0.60), the diagnostic odds ratio was 17 (95% CI: 4–77), and the area under the SROC curve was 0.87 (95% CI: 0.84–0.90) (Figures 3(a) and 3(b)).

### 3.4. Discussion

The results of our meta-analysis revealed a high diagnostic detection rate of polyps and BVNs by OCTA in PCV, and the detection rate of BVNs was higher than that of polyps. Our study also revealed a high combined sensitivity and specificity for the diagnosis of PCV from wAMD, suggesting that OCTA has acceptable diagnostic accuracy in diagnosing PCV.

Among the included studies, PCV polyps and BVNs showed different manifestations on OCTA images. Inoue et al. [26], Tomiyasu et al. [22], Wang et al. [23], and Mao et al. [14] observed that BVNs were located between Bruch's membrane and RPE, showing slightly higher flow signals of branch vessels, which can be distinguished from the surrounding normal blood vessels. However, Chi et al. [18] reported that some BVNs were located above or below the RPE. Tanaka et al. [16] classified PCV and found that all BVNs in type 1 PCV (polypoidal CNV) were situated between the RPE and Bruch's membrane, while BVNs in 64% of type 2 PCV (typical PCV) were visible only in the choroid layer. Wang et al. [23] noted that the BVN is more clearly imaged on OCTA than ICGA and can be divided into medusa, seafan, and tangled patterns according to the BVN morphology, whereas Huang et al [29] divided the BVN into trunk, glomeruli, and stick patterns. But in general, BVNs were manifested as high flow signals on OCTA and can be identified easily. Various manifestations of polyp lesions in PCV were also reported in the included studies in which we found high heterogeneity and publication bias according to Egger's test and the funnel plot (see [Supplementary-material supplementary-material-1]). Rebhun et al. [15] reported that polyps were located in the center of the BVN, presenting as low-signal nodules surrounded by high-signal vessels. However, Cheung et al. [10], Peiretti et al. [12], and Kim et al. [24] observed that polyps could exhibit either high or low flow signals, where the ratios of high signals were 41.7%, 83.3%, and 50%, respectively. In this regard, some scholars have provided explanations. Cheung et al. [10] observed unequally distributed blood flow signals in a polyp on different OCTA cross-sections. de Carlo et al. [11] hypothesized that polyps may be too small to be detected on OCTA or covered by BVN blood flow signals, resulting in an unclear polyp structure.

Our study also concluded that the detection rate of BVNs was higher than that of polyps with the use of OCTA in the diagnosis of PCV. The reason may be related to the different blood flow velocities of the lesion. Chi et al. [18] hypothesized that the structure of a polyp is the dilation of the end of BVN vessels in PCV lesions. Due to the larger diameter of the vessels, the blood flow velocity is slower inside the polyp, and when it is below the OCTA detection threshold, it cannot be seen on OCTA. In contrast, the surrounding BVN comprises the capillary vessels derived from the choroid with fast blood flow, which can be easily identified on OCTA.

Our study also showed that the application of OCTA to diagnose PCV among patients with wAMD had an acceptable diagnostic value. In a study by Cheung et al. [21], the diagnostic sensitivity of using OCTA to detect polyps was lower than that to detect BVNs, but the specificity was higher in polyps than in BVNs; therefore, polyps may be a more specific indicator of the diagnosis of PCV. Huang et al. [17] proposed that if an orange-red nodular lesion was seen on the fundus color photograph and OCT showed a double-layer sign or finger-like protrusion, the diagnosis of the eye would be “possible PCV”; if both polyps and BVN structures could be found on OCTA, “PCV” could be diagnosed; and if only the BVN was observed on OCTA and no polyps were seen, an ICGA exam is necessary to confirm PCV. This theory is also consistent with the findings of our study. Therefore, in the diagnosis of PCV from wAMD, if the BVN was obviously detected in OCTA, further polyp detection in OCTA was a strong sign for a diagnosis of PCV; if not, further ICGA was required for cases without BVN or further polyp in OCTA.

### 3.5. Limitations

Our meta-analysis had some limitations: First, the number of cases included in this study is limited because the incidence of PCV in the population is low, and OCTA is a new ophthalmology technology that is gradually being promoted in clinical settings. Second, OCTA detection rates in the diagnosis of the PCV polyps and BVNs are heterogeneous in our reports. Although we attempted to use subgroup analysis by the segmentation of OCTA, using SD-OCT or SS-OCT, and whether the participants received treatment, no significant differences were found in the results. The source of heterogeneity may be derived from the limited number of included studies, various experience of doctors, or whether lesion detection was combined with other imaging methods. Third, treated PCV was included in our study because of the limited number of studies that purely included treatment-naïve PCV and limited advanced evidence concerning whether treated PCV decreased the diagnostic efficacy of OCTA in the examination of PCV. Finally, the number of diagnostic tests is limited. More research is needed to explore the clinical value of OCTA in the diagnosis of PCV.

## 4. Conclusions

The present meta-analysis demonstrated that OCTA has a high detection rate for PCV polyps and BVN lesions, and the detection rate of BVNs is higher than that of polyps. OCTA has acceptable sensitivity and specificity for diagnosing PCV from wAMD in patients. Thus, OCTA is helpful for the clinical diagnosis of PCV.

## Figures and Tables

**Table 1:** Characteristics of included studies.

Study	Type of study	Diagnostic test	OCTA	Type of OCTA	Scanning range	Segmentation	Country	Inclusion criteria	Exclusion criteria	Age	Male (%)	PCV (ICGA) (*n*)	Treated PCV (%)
Huang et al. [29]	Retrospective	Y	Optovue AngioVue	SD	3 × 3	Manual	Taiwan, China	The diagnosis of PCV by ICGA and received OCTA simultaneously	Polyps beyond a 3 × 3 mm area from the central macula, other causes of fundus neovascularization	67.5 ± 7.3	68.00	50	42.0
Cheung et al. [1]	Prospective	Y	OCT (Spectralis; Heidelberg), OCTA only (Topcon DRI OCT Triton), OCT + OCTA	SS	6 × 6	Manual	Singapore	53 consecutive patients who presented with treatment-naive exudative AMD	Choroidal neovascularization secondary to causes other than AMD	69.5 ± 8.14	65.20	23	0.0
de Carlo et al. [11]	Retrospective	Y	Carl Zeiss Meditec Angioplex	SD	NA	Manual	Hawaii	Eyes with AMD (both PCV and non-PCV subtypes) with structural en face OCT or OCTA imaging	Eyes with concomitant retinal diseases, previous focal laser, major trauma, or intraocular surgery, AMD lesions outside of the imaging area	NA	87.50	41	NA
Peiretti et al. [12]	Retrospective	N	Optovue AngioVue	SD	3 × 3	Manual	Italy	PCV secondary to CSC based on clinical and multimodal imaging (FP, FFA, ICGA)	Relevant opacities of the optic media, low-quality images obtained with OCTA, previous treatments, presence of other concomitant ocular diseases, poor-quality images	67.35 ± 11.96	55.00	20	0.0
Chan et al. [13]	Prospective	N	Optovue AngioVue	SD	3 × 3/6 × 6	Auto	China	Confirmation of the diagnosis of PCV	Detectable BVNs without any polypoidal lesion detected on the ICGA, images of OCTA with a strength signal index lower than 50	61.1 ± 7.6	61.29	32	0.0
Mao et al. [14]	Retrospective	N	Optovue AngioVue	SD	3 × 3/6 × 6	Manual	China	Elevated orange-red lesions on fundus examination, and/or polypoidal lesions on ICGA	Poor-quality OCT images due to cataract or poor fixation, choroidal neovascularization secondary to causes other than AMD	NA	NA	14	NA
Lin and Shi [30]	Retrospective	N	Optovue AngioVue	SD	NA	NA	China	Orange-red lesions on fundus examination, and/or polypoidal lesions on ICGA	Relevant opacities of the optic media, low-quality images obtained with OCTA, a history of trauma, previous treatments, presence of other concomitant ocular diseases	NA	NA	48	NA
Rebhun et al. [15]	Prospective	N	Prototype SS-OCT + OCTA VISTA	SS	3 × 3/6 × 6	Manual	New England	Confirmed diagnosis of PCV by ICGA	NA	71 ± 10	28.57	7	14.3
Takayama et al. [27]	Prospective	N	Optovue AngioVue	SD	3 × 3	Auto	Japan	Early subretinal ICGA hyperfluorescence	Eyes with >25 and <22 mm axial length, polyp outside of the scanning area, any other retinal pathology, previously been treated, unclear images	73.8 ± 9.8	71.43	21	0.0
Huang et al. [17]	Retrospective	N	Optovue AngioVue	SD	NA	Manual	Taiwan, China	Presence of polyps with or without BVNs on ICGA	History of treatment including PDT or intravitreal injections of anti-VEGF therapy	NA	NA	31	0.0
Chi et al. [18]	Prospective	N	Optovue AngioVue	SD	3 × 3	Manual	Taiwan, China	Polyp-like choroidal vessel dilatation with a BVN on ICGA	Affected by any other macular disorders, and a history of other macular abnormalities related to diabetic retinopathy, retinal vein occlusion, uveitis, etc.	68.9 ± 8.0	NA	47	48.9
de Carlo et al. [19]	Retrospective	N	Carl Zeiss Meditec Angioplex	SD	NA	Manual	Asian, Filpino, Caucasian	Diagnosed with PCV using ICGA	Eyes with other concomitant retinal diseases including diabetic retinopathy, artery and vein occlusion, and macular telangiectasia	77 (53–92)	51.06	47	85.1
Xu and Lin [20]	Retrospective	N	Angio-retina	SD	6 × 6	Manual	China	Elevated orange-red lesions on fundus examination, and/or polypoidal lesions on ICGA	Combined with pathologic myopia, or a history of surgery or trauma	66.03 ± 8.14	70.00	20	NA
Tanaka et al. [16]	Retrospective	N	Optovue AngioVue	SD	3 × 3/6 × 6	Manual	Japan	Presence of polypoidal lesions on ICGA, categorized into polypoidal CNV (type 1 PCV) or typical PCV (type 2 PCV)	NA	NA	75.00	32	0.0
Cheung et al. [21]	Prospective	Y	Topcon DRI OCT Triton	SS	3 × 3	Manual	Singapore	Diagnosis of PCV based on clinical examination and underwent FFA and ICGA	NA	68.89 ± 9.41	63.00	54	68.5
Tomiyasu et al. [22]	Retrospective	N	Optovue AngioVue	SD	6 × 6	Manual	Japan	Diagnosis of treatment-naive PCV, based on ophthalmoscopic examinations, ICGA, and OCT	Poor-quality OCT images due to cataract or poor fixation	71.85 ± 9.53	90.00	20	0.0
Wang et al. [23]	Retrospective	N	Optovue AngioVue	SD	3 × 3	Manual	China	Diagnosis of PCV by FP, FFA, ICGA, SD-OCT	A large area of hemorrhage or cloudy media that could significantly reduce the intensity of the OCTA signal	59.3 ± 5.43	84.62	13	15.4
Kim et al. [24]	Retrospective	N	Optovue AngioVue	SD	3 × 3/6 × 6	Manual	Korea	Hyperfluorescent polyps detected by ICGA	Cases accompanied by severe subretinal hemorrhage and scarring show inaccurate segmentations	67.86 ± 14.02	71.43	7	71.4
Srour et al. [25]	Prospective	N	Optovue AngioVue	SD	3 × 3	Auto	France	Diagnosis of PCV based on FP, FFA, ICGA, SD-OCT	Affected with any other macular disorders such as high myopia (>8 diopters), presence of angioid streaks, or intraocular inflammation	72.6 ± 10.5	33.33	12	83.3
Inoue et al. [26]	Retrospective	N	Optovue AngioVue/Heidelberg Spectralis OCTA	SD	3 × 3	Manual	US	Demonstrated polypoidal changes to neovascular tissue on ICGA and SD-OCT	NA	71.1 ± 10.9	14.29	7	85.7
Ma et al. [28]	Retrospective	N	Optovue AngioVue	SD	6 × 6	Auto	China	Elevated orange-red lesions on fundus examination/polypoidal lesions on ICGA	Combined with pathologic myopia, idiopathic choroidal neovascularization, history of surgery or trauma, low vision that cannot complete OCTA exam	68.24 ± 6.80	52.94	17	0.0

OCTA: optical coherence tomography angiography; SD: spectral domain; SS: swept source; PCV: polypoidal choroidal vasculopathy; ICGA: indocyanine green angiograph; FFA: fundus fluorescein angiography; FP: fundus photograph; wAMD: wet age-related macular degeneration; PDT: photodynamic therapy; VEGF: vascular endothelial growth factor; CNV: choroidal neovascularization; NA: not available; Y: yes; N: no.

## Data Availability

The pooled analysis data used to support the findings of this study are available from the corresponding author upon request.

## Supplementary Materials

Supplementary Materials Supplementary Figure 1 presents the methodological quality graph of included studies. Supplementary Figure 2 shows the funnel plots of the combined detection rate. Supplementary Figures 3 and 4 present subgroup analysis of polyp and BVN detection rates.
