# Supplementary figures and images for: Detection Rate and Diagnostic Value of Optical Coherence Tomography Angiography in the Diagnosis of Polypoidal Choroidal Vasculopathy: A Systematic Review and Meta-Analysis
Source: J Ophthalmol. 2019 Dec 14;2019:6837601. doi: 10.1155/2019/6837601 (PMC6931027; doi:10.1155/2019/6837601)

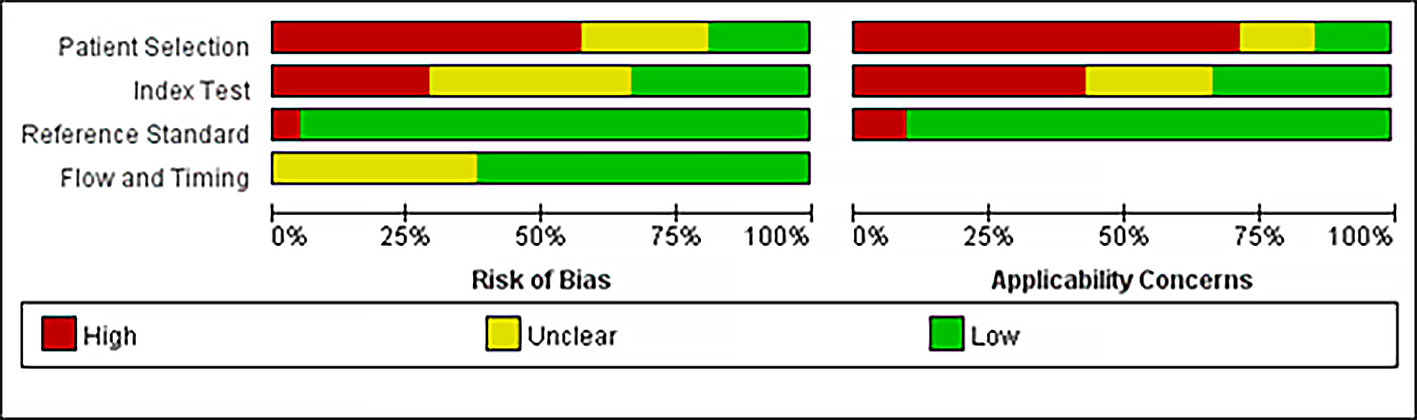

Supplement: Supplementary Materials — Supplementary Figure 1 presents the methodological quality graph of included studies. Supplementary Figure 2 shows the funnel plots of the combined detection rate. Supplementary Figures 3 and 4 present subgroup analysis of polyp and BVN detection rates. [file 6837601.f1.zip › 6837601.f1/Figure S1.tif]

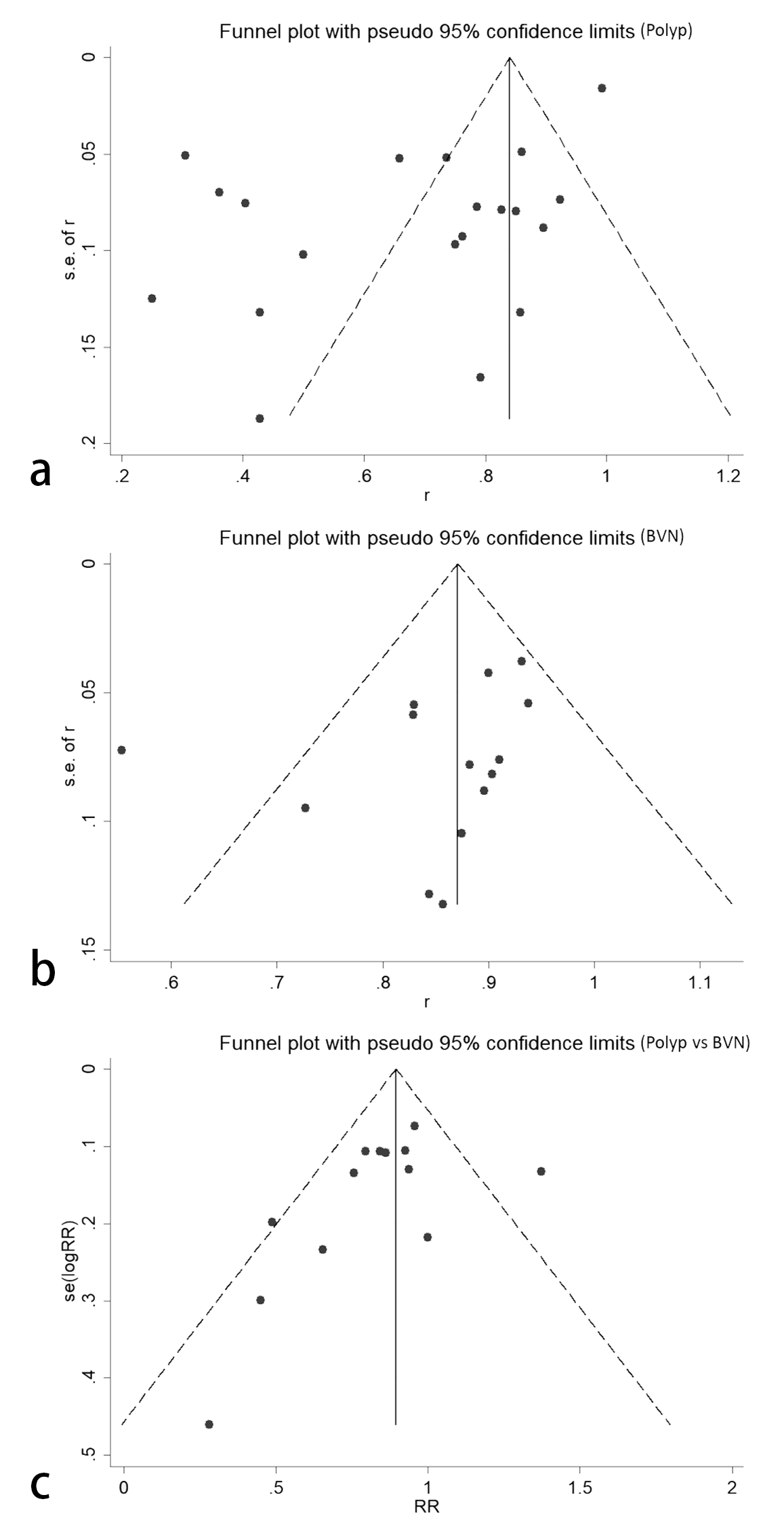

Supplement: Supplementary Materials — Supplementary Figure 1 presents the methodological quality graph of included studies. Supplementary Figure 2 shows the funnel plots of the combined detection rate. Supplementary Figures 3 and 4 present subgroup analysis of polyp and BVN detection rates. [file 6837601.f1.zip › 6837601.f1/Figure S2.tif]

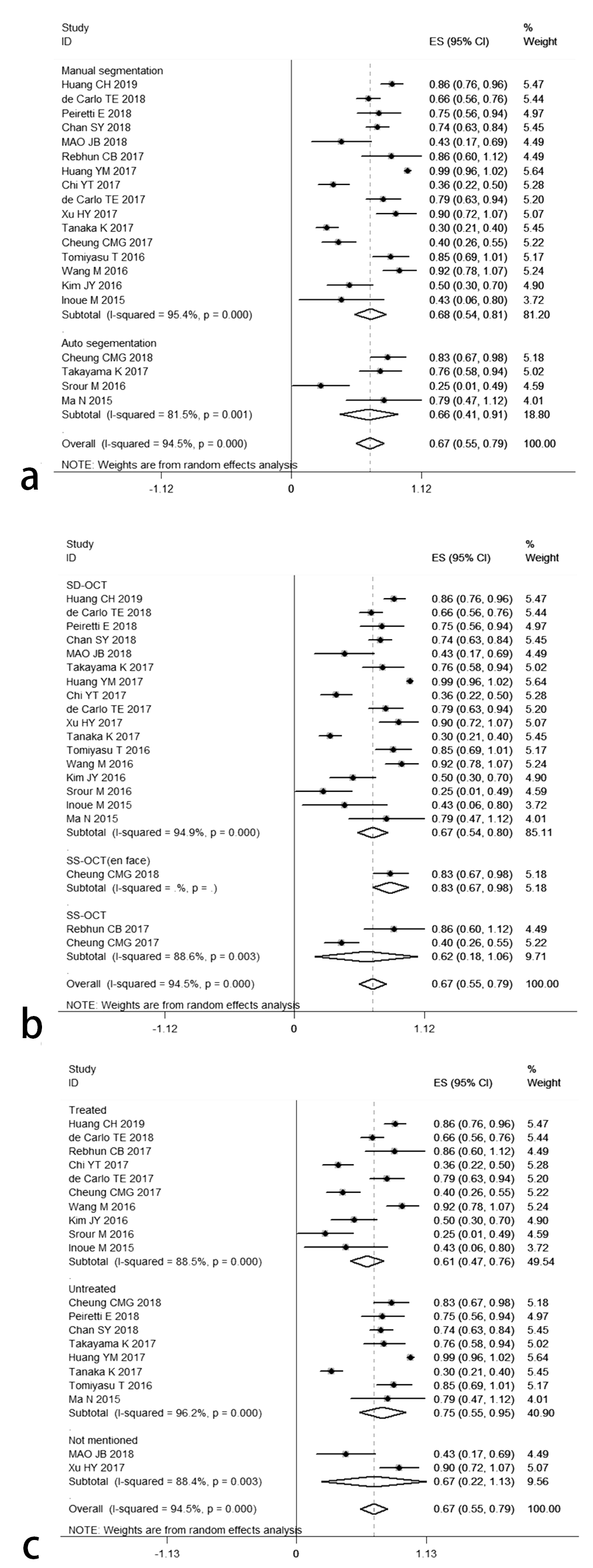

Supplement: Supplementary Materials — Supplementary Figure 1 presents the methodological quality graph of included studies. Supplementary Figure 2 shows the funnel plots of the combined detection rate. Supplementary Figures 3 and 4 present subgroup analysis of polyp and BVN detection rates. [file 6837601.f1.zip › 6837601.f1/Figure S3.tif]

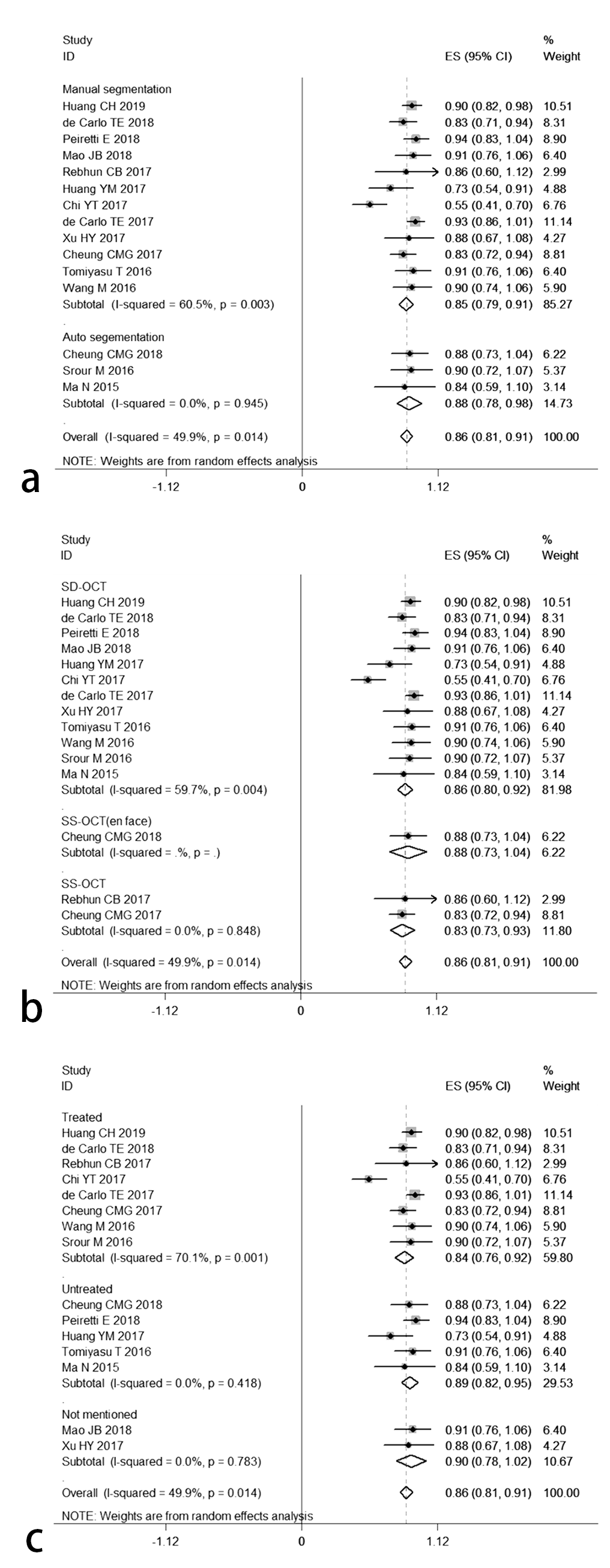

Supplement: Supplementary Materials — Supplementary Figure 1 presents the methodological quality graph of included studies. Supplementary Figure 2 shows the funnel plots of the combined detection rate. Supplementary Figures 3 and 4 present subgroup analysis of polyp and BVN detection rates. [file 6837601.f1.zip › 6837601.f1/Figure S4.tif]
